# Association of gestational hypertension and preeclampsia with offspring adiposity: A systematic review and meta-analysis

**DOI:** 10.3389/fendo.2022.906781

**Published:** 2022-08-23

**Authors:** Shiyu Yan, Jinlang Lyu, Zheng Liu, Shuang Zhou, Yuelong Ji, Haijun Wang

**Affiliations:** ^1^ School of Public Health, Zhejiang University Medical School, Hangzhou, China; ^2^ Department of Maternal and Child Health, School of Public Health, Peking University, National Health Commission Key Laboratory of Reproductive Health, Beijing, China

**Keywords:** gestational hypertension, preeclampsia, maternal, offspring, adiposity

## Abstract

**Background:**

The association of gestational hypertension (GH) and preeclampsia (PE) with offspring adiposity outcomes had controversial results in different studies.

**Objective:**

We conducted a systematic review and meta-analysis to evaluate the relationship between maternal GH/PE and offspring adiposity outcomes.

**Search strategy:**

Studies were identified in PubMed, Embase, and Cochrane databases, with keywords including “gestational hypertension”, “preeclampsia”, “offspring”, “weight”, “cohort study”, etc., without year restriction. This study was registered with PROSPERO, CRD42022292084.

**Selection criteria:**

We set the selection criteria for six aspects: population, outcome, time frame, study design, and availability. For the studies included in the meta-analysis, we required the potential confounders in these studies have been adjusted.

**Data collection and analysis:**

Two reviewers independently evaluated the data from the included studies. The meta-analyses included mean differences, regression coefficients, and corresponding 95% confidence intervals. Results were performed using RevMan software (version 5.4; Cochrane Collaboration). Heterogeneity among the included studies was assessed using the I^2^ statistic.

**Main results:**

A total of 16 studies were included in our review, 15 of which were evaluated as high quality. In all offspring, during the early life (28 days-36 months), GH/PE exposure was found to be not or inversely associated with offspring obesity, then become positively associated at larger ages (3-19 years old). In offspring with adverse birth outcomes, the maternal GH/PE-exposed group had a lower weight in the short term (28 days to 18 months), but there was a trend of rapid weight gain as they grew older, compared with the non-exposed group. The meta-analysis showed that the BMI of the female offspring in the maternal PE-exposed group was significantly higher than that of the non-exposed offspring (MD=1.04, 95% CI: 0.67~1.42, *P* < 0.05).

**Conclusions:**

The systematic review suggested that maternal exposure to *de novo* hypertension disorders of pregnancy (HDP) was associated with obesity in offspring, extending from early childhood to adolescence. The meta-analysis showed that PE was associated with higher BMI in female offspring. More studies are needed to conduct stratified analyses by PE/GH, the severity of HDP, or gender.

**Systematic review registration:**

PROSPERO, identifier CRD42022292084.

## Introduction

Obesity in children and adolescents has become one of the most vital public health problems due to its high prevalence and its adverse outcomes. According to the data from World Health Organization, there were only less than 1% of children aged 5-19 with obesity in 1975, but 38.2 million children under 5 and over 340 million children and adolescents aged 5-19 were with overweight or obesity in 2019 (1). Childhood obesity would have both short- and long-term health effects, such as increased asthma risk, musculoskeletal problems, and cardiovascular disease in adulthood (2–4). Meanwhile, studies have shown that childhood obesity could persist into adulthood, which indicated the importance of preventing obesity at an early stage (5).

The influencing factors of childhood obesity, like nutritional factors, physical activity, sleep status, and family factors, mainly focused on the different stages of life after childbirth. However, more evidence showed that the origins of childhood obesity can be as early as maternal pregnancy. According to “Developmental Origins of Health and Disease Hypothesis” (DOHaD), a lack of nutrients in the uterus would cause epigenetic changes and evolutionary adaption of the offspring. After childbirth, the catch-up growth of offspring would have short-term survival benefits but permanent alterations in the body’s metabolism (6–9). Both animal experiments and epidemiological studies have shown a significantly increased prevalence of obesity in the group exposed to adverse pregnancy factors than in the controls (10–12).

Hypertension disorders of pregnancy (HDP) may be one of the adverse pregnancy factors mentioned above. HDP complicates about 5–10% of pregnancies, contributing to severe maternal and fetal health outcomes (13). It can be divided into four categories: gestational hypertension (GH), preeclampsia (PE)/eclampsia, chronic hypertension (CH), and CH with superimposed PE (14). Different from pre-existing hypertension with or without PE, both GH and PE are characterized by *de novo* HDP. GH occurs at any time after 20 weeks of pregnancy, and if proteinuria (excretion of protein over 300 mg per day) occurs the disease was thought to develop into PE. In some cases, PE could occur without a prior diagnosis of GH. In 2017, a review of epidemiological studies reported the global prevalence of GH and PE were 1.8–4.4% and 0.2–9.2%, respectively (15).

Some studies have investigated the relationship between maternal HDP and offspring adiposity outcomes (16–18). However, since CH and *de novo* HDP may have different mechanisms, combining all types of HDP would cause bias (19, 20). Furthermore, most previous studies focused on adult offspring (21), and there were a few studies focused on obesity of offspring in children and adolescents, in which the findings were inconsistent (21, 22). Only two systematic reviews in the past have discussed the relationship between HDP and offspring adiposity outcomes, but there were limitations such as unclear classification of HDP, and less specific inclusion criteria (23, 24). In light of these inconsistent results and the limitations of previous systematic reviews, it is necessary to conduct a systematic review focusing on *de novo* HDP and offspring adiposity outcomes.

We conducted the systematic review aiming to systematically review and quantitatively analyze the current evidence on the association between *de novo* HDP and offspring adiposity outcomes.

## Methods

We carried out this systematic review based on the recommendations of the Preferred Reporting Items for Systematic Review and Meta-Analysis (PRISMA) guidelines. The protocol of this review was registered in the PROSPERO database (registration number: CRD42022292084).

### Data sources and search strategy

We performed a literature search within the PubMed, Embase, and Cochrane databases without year restriction. The search strategy was defined according to the “PICOS” principle: “P”-offspring, “I”-exposed to GH/PE, “C”-non-exposed to GH/PE, “O”-adiposity outcomes,”S”-cohort study. Thus, the searches were conducted using Medical Subject Heading terms and the keywords including (“Pregnancy” OR “Maternal”) AND (“Pre-Eclampsia” OR “Preeclampsia”) AND (“Hypertension, Pregnancy-Induced” OR “Gestational Hypertension” OR “Pregnancy Induced Hypertension”) AND (“Infant, Newborn” OR “Adolescent” OR “Pediatrics” OR “offspring”) AND (“Body Weight” OR “adiposity” OR “adipose” OR “body mass index” OR “obesity” OR “waist circumference”) AND (“cohort” OR “retrospective” OR “prospective” OR “follow up”). The detailed search strategy is found in the **Supplementary Table 1**. Then we used the snowball method to manually scrutinize the reference lists of the identified articles, looking for the relevant studies.

### Selection criteria

Two reviewers (SY and JL) independently evaluated the studies according to the following criteria (**Table 1**).

**Table 1:** Selection criteria for studies included in the review.

	Inclusion Criteria	Exclusion Criteria
**Population**	●Mothers with antenatal exposure to PE or GH	●Including other pregnancy-induced hypertension without differentiation (e.g. eclampsia)
**Outcome**	●Mainly investigating the association between PE/GH and indicators relevant to offspring adiposity outcomes (including body weight, BMI, BMI Z score, etc.) ●Offspring aged 0-18 (We also included a study in which the offspring was aged 13-19 because most populations meet the requirement.)	●Focusing on other indicators of offspring adiposity outcomes not related to adiposity. ●Offspring over 18 years of age
**Time frame**	●Any time frame	
**Study design**	●Human studies ●Cohort studies (prospective cohort study, retrospective cohort study, birth cohort, follow-up of the nested case-control study) ●Having a control group (women with normotensive pregnancy)	●Randomized Controlled Trial ●Review ●Editorial ●Case report ●Commentary article ●Animal studies
**Availability**	●Able to find the full-text article ●Published in English	●Non-English language

Abbreviations: PE, preeclampsia; GH, gestational hypertension; BMI, body weight index.

Studies were included in the meta-analysis if they met the following criteria:

(1) There were available data: mean differences (MD), regression coefficient (β), and their corresponding 95% confidence intervals (CI).
(2) Potential confounders were adjusted.

### Data extraction and quality assessment

Two reviewers (SY and JL) independently screened the titles and abstracts according to the inclusion and exclusion criteria. For studies potentially eligible for the requirements, full texts were obtained to screen. Then the data from all potentially eligible studies were extracted with a predefined data extraction form (an Excel sheet). The following information was extracted: name of the first author, year of publication, study location, ethnicity, the sample size of the control group (without PE/GH), the sample size of the PE/GH group, the definition of PE/GH, follow-up time, evaluation of the offspring adiposity outcomes, and adjusted potential confounders. Any disagreements would be resolved by discussion. And when consensus could not be reached, disagreements were settled by a third reviewer (ZL). Quality evaluation was assessed using the Newcastle–Ottawa Quality Assessment Scale (NOS). A maximum of nine stars can be given to one study. Two reviewers independently carried out the assessment. The study with a NOS score ≥7 was considered high quality. When 5 ≤ NOS score ≤ 7, the study was considered as moderate quality. And when a NOS score was below 5, the study quality was considered as low.

### Statistical analysis

In the meta-analysis, the study included the results of obesity-related indicators with adjustment for confounding factors, including MD, β and corresponding 95% CI. The analysis was performed using RevMan software (version 5.4; Cochrane Collaboration). Heterogeneity among the included studies was assessed using the I^2^ statistic. When I^2^ >50%, there was heterogeneity among studies and a random-effect model was used; When I^2^ ≤50%, the heterogeneity between studies was low and a fixed-effect model was used.

## Results

### Search results

**Figure 1:**
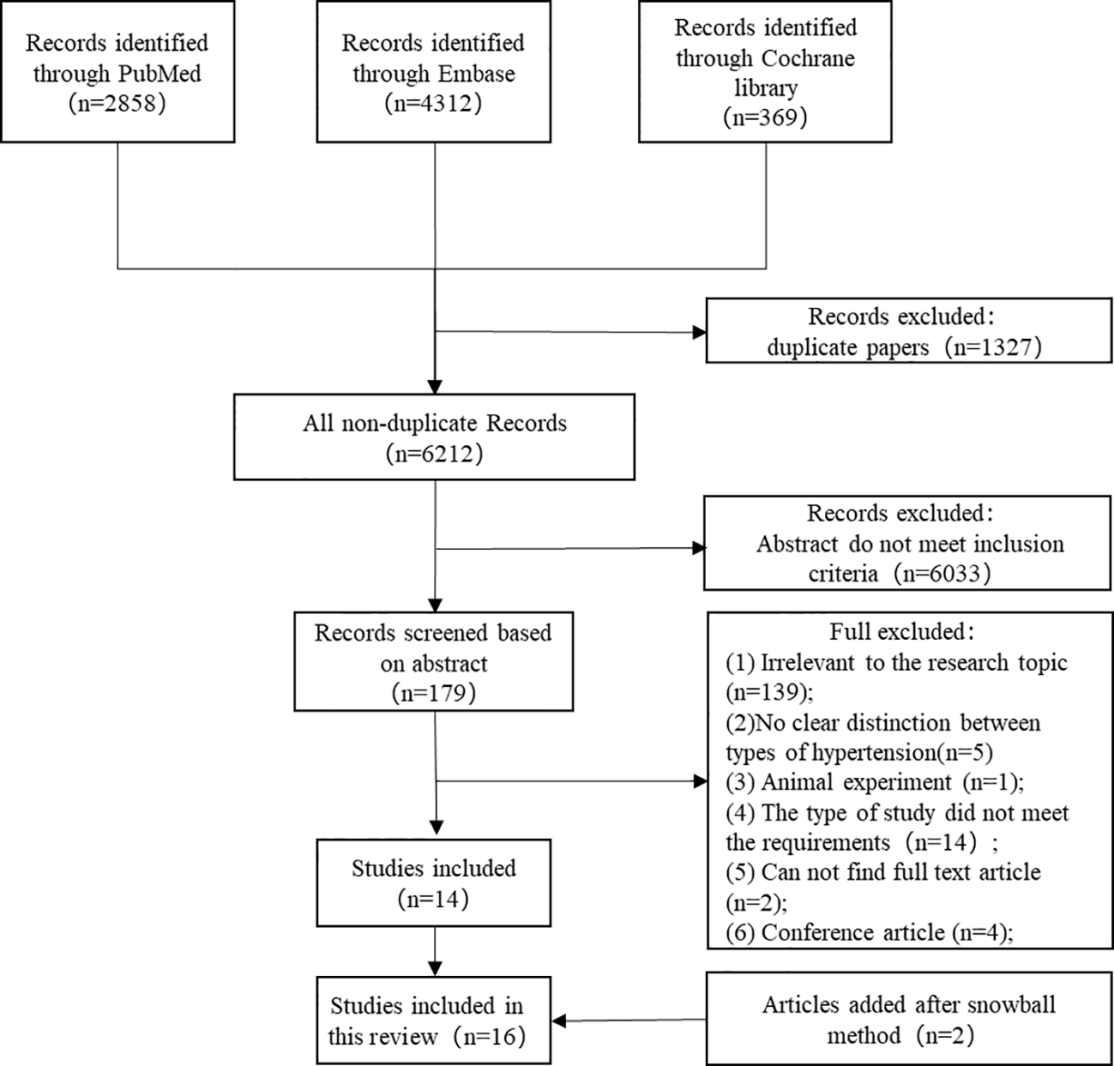
Flow chart of the literature search and selection of studies.

The flow chart of the literature search and selection of studies was shown in **Figure 1**. A total of 6,212 titles were retrieved from the search, only 14 met all selection criteria and were included in this systematic review. Two additional articles were incorporated after using the snowball method to search through the reference lists of identified articles. Finally, a total of 16 articles were included in our review. Important characteristics (author/year, sample size and grouping, observes age, a summary of results, control of confounding factors, and NOS) of these studies were shown in **Table 2**.

**Table 2:** Important characteristics of the included studies.

No	Author/year	Sample size and grouping	Observed age	Summary of results	Control of confounding factors	NOS
1	Baulon E et al., 2005	Control 10069; GH 782; PE 365; Severe PE/eclampsia 105;	28 and 42 days after delivery	28 days Infant Growth Percentage, β (SE) [[(infant weight - birth weight)/birth weight] × 100%] GH/non-IUGR -4.4 (18.6); PE/non-IUGR 38.0 (25.8); 42 days Infant Growth Percentage, β (SE) GH/non-IUGR -12.8 (22.8); PE/non-IUGR 55.4 (33.5);	Maternal age, body mass index, diabetes, infant’s sex, maternal anemia, gestational age	7
2	Megan L Gow et al. 2021	Control 298; PE 84;	0-6 months	6month Weight (kg), mean (SD) Non-PE 7.93 (0.96); PE7.61 (0.99)* ; BMI (kg/m^2^), mean (SD) Non-PE 16.89 (1.50); PE 16.64 (1.46); Weight Z score, mean (SD) Non-PE 0.05 (0.94);PE -0.21 (0.95)*; Weight gain(kg), mean (SD) Non-PE 4.56 (0.94);PE 4.86 (0.99)*;	Crude results	9
3	Silveira RC et al., 2007	Control 40; PE 46;	12 months and 18 months corrected ages	At 12 months corrected age Weight (g), mean ± SD Non-PE 9223 ± 231; PE 8610 ± 120*; Weight/age Z, mean ± SD Non-PE −0.71 ± 1.36; PE−1.36 ± 0.88*; Weight/length Z, mean (SD) non-PE −0.37 ± 1.34; PE −1.08 ± 1.22*; At 18 months corrected age Weight (g), mean ± SD Non-PE 12510 ± 139; PE 9785 ± 142*; Weight/age Z, mean ± SD Non-PE −0.88 ± 1.17; PE −1.48 ± 0.97; Weight/length Z, mean (SD) non-PE −0.66 ± 1.24; PE−1.37 ± 1.09**;	Crude results	9
4	Jiang W et al., 2021	Normal 31171; GH408;	1, 3, 6, 8, 12, 18, 24, 30, 36 months	Weight, β(SE) GH - 0.15 (0.05)**; Weight-for-age Z score, β(SE) GH -0.05 (0.02)*;	Offspring sex, maternal age at delivery, maternal pre-pregnancy body mass index, parity, educational level, birthweight	9
5	Randhir K et al., 2020	Control 470; PE 681;	3-7 years old	Weight Z score, β (95%CI) PE 0.21 (0.059, 0.48)*; BMI Z score, β (95%CI) PE: 0.13 (-0.09, 0.36);	Birth weight, gestational age, maternal BMI, maternal height, and SLI score	9
6	Palti H et al. 1989	Control 94; PE94;	6 years old	Weight (kg) (male), mean(SD) Non-PE19.2 ± 3.6; PE 20.1 ± 3.4; Weight(kg) (female), mean(SD) Non-PE 18.8 ± 3.0; PE 19.1 ± 3.5;	Crude results	6
7	Huang Y et al., 2020	Control 332; PE 24;	18-72 months	Hierarchical Linear Modeling: BMI trajectory by preeclampsia increased over time (*t* ratio=3.153, β=0.65, 95% CI 0.11-1.18, *p* = 0.002)		8
8	Geelhoed JJ et al., 2017	Normal 5345; GH1118; PE205;	9 years old	BMI z score, β (95% CI) GH 0.05 (-0.02–0.12); PE -0.19 (-0.34–0.05) Waist circumference z score, β (95% CI) GH 0.04 (-0.03–0.10); PE -0.20 (-0.34–0.05); Fat mass z score, β (95% CI) GH 0.04 (-0.03–0.10); PE -0.09 (-0.23–0.05); Lean mass z score, β (95% CI) GH 0.01 (-0.04–0.05); PE -0.19 (-0.28–0.09); Obese, OR (95% CI) GH 1.37 (0.99–1.89); PE 0.50 (0.21–1.20); Overweight or obese, OR (95% CI) GH 1.08 (0.90–1.30); PE 0.60 (0.39–0.93); Central obesity, OR (95% CI) GH 1.04 (0.89–1.21); PE 0.81 (0.57–1.15);	Offspring sex and age, maternal age at delivery, parental pre-pregnancy BMI, parity, social class, and maternal smoking during pregnancy, plus offspring weight, height, and height squared at the 9-year visit	9
9	Palma Dos Reis CR et al., 2021	Normal 5066; PE 67;	10 years old	BMI Z score, β (95%CI) PE -0.014 (−0.300, 0.272); Overweight/obesity status, OR (95% CI) PE 1.23 (0.74, 2.03);	BMI Z score: Pre-pregnancy BMI, primipara status, and tobacco smoke during pregnancy Overweight/obesity status: pre-pregnancy BMI, multipara status	9
10	Ogland B et al., 2009	Control: 194 pairs of mother and daughter and 166 pairs of mother and son; PE: 91 pairs of mother and daughter and 92 pairs of mother and son;	Girl: 10.8 years old; Boy: 11.8 years old;	BMI (female), mean PE 18.4; non-PE 17.5; Difference (95% CI) 0.96 (0.2, 1.7)*;	weight for gestational age (z score)	9
11	Aris IM et al., 2018	Normal 1194; GH 97; PE 45	From birth to 131.2 months	Predictors of age at BMI peak and rebound (in months) from stepwise regression analyses, β (95%CI) GH -0.1(-0.6, 0.5); PE 1.6(0.8, 2.4);		9
12	Byberg KK et al., 2017	Control 385; PE 229 (mild/moderate: 164, 54)	13 years old	weight SDS, β (95%CI) mild/moderate PE 0.08(-0.16,0.33); severe PE -0.19(-0.59, 0.21); BMI, β (95%CI) mild/moderate PE 0.21(-0.01,0.44); severe PE 0.14 (-0.24, 0.52); Waist-to-height ratio, β (95%CI) mild/moderate PE -0.16 (-0.06,0.38); severe PE 0.47 (0.15,0.79);	Child’s sex, Birth order, Maternal BMI, Maternal smoking in pregnancy, maternal age at delivery, maternal education at the time of deliver	9
13	Washburn L et al., 2013	Normal 121; PE 51	14 years old	BMI (kg/m^2^), mean differences (95%CI) Male adolescent 1.0 (-0.7, 2.7); Female adolescent -0.4 (-2.1, 1.3); Abdominal circumference (cm), mean differences (95%CI) Male adolescent 2.6 (-2.4, 7.5); Female adolescent -1.9 (-6.1, 2.3); Triceps skinfold thickness (mm), mean differences (95%CI) Male adolescent -0.2 (-3.7,3.4); Female adolescent 1.5 (-1.3, 4.4); Subscapular skinfold thickness(mm), mean differences(95%CI) Male adolescent 0.4 (-3.8, 4.6); Female adolescent 1.4 (-2.5, 5.3); Percent body fat, mean differences (95%CI) Male adolescent -0.3 (-4.5, 3.9); Female adolescent -0.3 (-3.1, 2.6);	Antenatal steroid exposure and race, birth weight z score, weight z score at 1-y corrected age–birth weight z score, weight z score at 14 y–weight z score at 1-y corrected age.	9
14	Miettola S et al. 2013	Control 5045; GH 331; PE 197	16 years old	Cholesterol (mmol/L), percentage difference (95% CI) GH 2.3 (0.2,4.4); PE 0.1 (-2.7,2.9); LDL (mmol/L), percentage difference (95% CI) GH 2.1 (-0.8, 5.1); PE -0.1 (-3.9, 3.9); HDL (mmol/L), percentage difference (95% CI) GH 2.0 (-0.3, 4.4); PE -0.3 (-3.4, 2.8); Triglycerides (mmol/L), percentage difference (95% CI) GH -3.6(-8.0, 1.1); PE -1.2(-7.4, 5.3);	Sex, nulliparity, maternal pre-pregnancy BMI and socioeconomic position, offspring BMI at age 16, offspring birth weight	9
15	Davidesko S et al., 2020	Control 243701; PE 10107	18 years old	Overweight and obesity rate, % PE 0.4; Non- PE 0.2**;	Crude	8
16	Vatten LJ et al., 2003	Normal 3486; PE 220	13-19 years old	Weight(kg), mean (95%CI) PE 62.5 (61.3,63.7); Non-PE 59.1 (58.8, 59.4) **; BMI (kg/m^2^), mean (95%CI) PE 22.6 (22.2, 23.0); Non-PE 21.5 (21.3, 21.6)**; Waist (cm), mean (95%CI) PE 72.6 (71.6, 73.6); Non-PE 70.5 (70.3, 70.8)**;	Birth weight, gestational age at birth, and age at survey attendance	9

GH, gestational hypertension; PE, preeclampsia; β, regression coefficient; SE, standard error; IUGR, intrauterine growth retardation; SD, standard deviation; BMI, body mass index; CI, confidence interval; SDS, standard deviation score; LDL, low-density lipoprotein cholesterol; HDL, high-density lipoprotein cholesterol.

*P<0.05; **P<0.01.

### Qualitative description

Among the 16 included studies, 15 were evaluated as high-quality using NOS criteria, except for one study with moderate quality published in 1989 (25). The detailed NOS scores were found in the **Supplementary Table 2**. Among 15 studies with high quality, 10 studies merely included the population exposed to PE, and 5 studies included people exposed to GH or PE. According to the anthropometry age of offspring, there were 4 studies in the age group of 0-5 years, one study in the age group of 6-9 years, and 8 studies in the age group of 10-19 years. In addition, one study conducted measurements in children aged 3-7 and one conducted at 1.5-6 years of age. For adverse birth outcomes, 4 out of 15 studies observed the association in offspring with the adverse birth outcome, including intrauterine growth retardation (IUGR), very low birth weight (VLBW), small for gestational age (SGA), and preterm birth. For the other 11 studies on all offspring, the outcomes were different, with 9 comparing the continuous adiposity variables, 3 comparing the obesity/overweight prevalence, and 2 exploring the growth trajectory. The adverse birth outcomes and adiposity outcome measurements were concluded in **Table 3**.

**Table 3:** The adverse birth outcomes and adiposity outcome measurements of the included studies.

Author	Adverse birth outcomes	Continuous variable		Categorical variable(rate)		Growth trajectory
		Univariate analysis	Multivariate analysis	Univariate analysis	Multivariate analysis	
Baulon E et al.	●	●	●			●
Gow ML et al.	●	●				●
Silveira RC et al.	●	●				
Jiang W et al.			●			
Randhir K et al.		●	●			
Huang Y et al.						●
Geelhoed JJ et al.		●	●		●	
Palma Dos Reis CR et al.		●	●		●	
Ogland B et al.		●	●			
Aris IM et al.						●
Byberg KK et al.			●			
Washburn L et al.	●	●	●	●		●
Miettola S et al.		●	●			
Davidesko S et al.				●		
Vatten LJ et al.			●			

### The analysis of all offspring

#### Continuous variable

During early life (28 days-36 months), maternal exposure to GH/PE was found to be not or inversely associated with offspring obesity. One study found that there was no difference in weight at 28 and 42 days postpartum between the exposed group and the control group in both univariate and multivariate analysis (26). The study by Gow ML et al. found that at 6 months, the weight, and weight Z score of offspring exposed to PE remained significantly lower compared with the control group, but there was a significant absolute increase in BMI and weight from birth to 6 months (27). In a study by Jiang W et al., both GH and PE were inversely associated with offspring’s weight and weight-for-age Z score from birth to the age of 36 months, but the associations became nonsignificant after adjusting for birth weight in PE-exposed infants (28).

However, GH/PE tended to be positively associated with offspring adiposity indicators at larger ages (3-19 years old). Four studies adjusted for potential confounders including birth weight, gestational age at birth, maternal BMI, maternal height, maternal smoking status, maternal age at delivery, maternal education, and other factors, and all found a positive association between *de novo* HDP and offspring adiposity indicators (weight, BMI, waist-to-height ratio SDS, total cholesterol, waist circumference, and hip circumference) at the age of 3-19 (22, 29–31).

There were 2 studies with inconsistent findings. In a study by Geelhoed JJ et al., for offspring aged 9 years, the previous positive association between GH and offspring adiposity outcomes became insignificant after adjusting for gestational weeks and birth weight, but the insignificant association between PE and adiposity outcomes (BMI, waist circumference, fat mass) became inverse once the maternal BMI was controlled for. The authors suggested GH/PE may be distinct conditions and that IUGR may be a mediator between PE and offspring obesity (32). Palma Dos Reis CR et al. also found no significant difference in BMI, or BMI *Z* score between PE-exposed 10-year-old offspring and the controls after adjusting for pre-pregnancy BMI and tobacco use. The authors suggested that maternal pre-pregnancy BMI, primiparity, and tobacco use were important confounders of PE and offspring obesity. However, as the authors pointed out, one of the reasons for the inconsistent results may be the limited number of PE cases (n=67) (33).

#### Categorical variable (overweight/obesity)

Three studies compared the overweight/obesity prevalence of offspring between the GH/PE-exposed and normotensive groups. In a study by Geelhoed JJ et al., a positive association of offspring overweight/obesity with GH was identified, but offspring exposed to PE were less likely to be obese at 9 years old compared with the normotensive group (32). Davidesko S et al. observed a higher prevalence of overweight and obesity in 18-year-old offspring born to mothers with PE (34). However, Palma Dos Reis CR et al. found that there was no association between PE and risk of obesity at 10 years old (33). The inconsistent results may be due to different sample sizes and study populations, as well as pathological differences between GH and PE, which needed to be further confirmed.

#### Growth trajectory

There were 2 studies evaluating the BMI trajectories of offspring and both found the growth trend related to obesity. Using mixed-effects models to fit BMI curves, Aris IM et al. indicated that PE-exposed children had a later age (1.8 months) at the peak of the BMI trajectory than the control group, which was associated with obesity later in childhood (35). For the offspring at larger age, the study by Huang Y et al. used hierarchical linear modeling and explored that the BMI trajectory of the PE group increased over time from 18 months to 6 years compared to the non-exposed group (36).

### Analysis of offspring with adverse birth outcomes

Baulon E et al. found that GH/PE-exposed offspring with IUGR had a significantly lower birth weight but higher infant growth percentage (defined as weight gain from birth to infancy divided by birth weight) at 28 and 42 days postpartum compared to the group without IUGR (26). The other study found that PE-exposed infants with SGA had significantly greater weight Z score gain compared with PE-exposed infants without SGA from birth to 6 months (27). And among PE-exposed and non-exposed infants with VLBW, Silveira RC et al. observed lower weight, weight/age Z score, and weight/length Z score at the 12- and 18-months corrected age in the PE-exposed group (37). Washburn L et al. focused on 14-year-old offspring born prematurely and found PE-exposed offspring had higher BMI, waist circumference, subscapular skinfold thickness, and triceps skin-fold thickness compared to offspring born prematurely after normotensive pregnancies, and the association still existed after adjusting for birth weight Z score. In addition, males with PE exposure showed more weight gain during infancy, and females with PE exposure showed more weight gain from 1-year corrected age to 14 years compared to the control group (38). The studies above suggested that GH/PE-exposed offspring with adverse birth outcomes had a lower weight in early life (28 days to 18 months), but there was a rapid weight gain as they grew older.

### Subgroup results

Three studies further discussed the influence of different types or severity of *de novo* HDP. In the study by Geelhoed JJ et al., GH and PE showed opposite effects on obesity among 9-year-old children (32). Another study indicated that offspring exposed to GH tended to have higher lipid levels, but no association was observed between PE and offspring lipid values, suggesting these two types of HDP may have different mechanisms underlying their effects on offspring growth (22). Byberg KK et al. classified PE into mild/moderate and severe groups and found that mild/moderate PE was positively associated with body weight and BMI at 13 years old, but severe PE was negatively associated with them (30). Overall, given the small amount of available evidence and the lack of consistency across studies, it is unclear whether there were differences in the effects of different types (PE/GH) or severity on offspring obesity.

Four studies discussed the different effects of *de novo* HDP between girls and boys. One study showed that, for adolescents aged 14 years who were born prematurely with VLBW, male offspring exposed to PE had higher levels of adiposity, but no significant association was found among females (38). Ogland B et al. found significantly higher obesity indicators in female offspring aged 10.8 years of mothers with both PE and obesity (BMI≥30) but found no significant results in male offspring aged 11.8 years. The association of PE with adiposity measurements was significantly different between girls and boys when testing for interaction by gender (39). The other 2 studies also tested and found the interaction effects of gender with GH/PE (30, 33). Although the results for each gender were inconsistent, significant interaction terms of GH/PE and gender suggested that *de novo* HDP may have gender-specific effects.

### Meta-analysis

**Figure 2:**
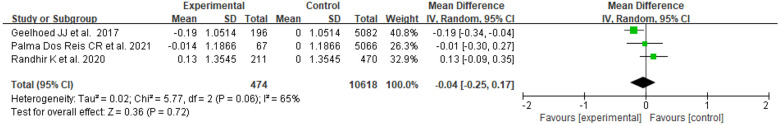
Forest plot for the mean difference in BMI Z score between PE-exposed and unexposed offspring.

**Figure 3:**
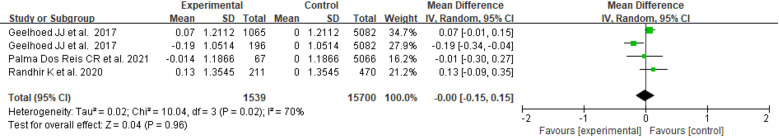
Forest plot for the mean difference in BMI Z score between GH- or PE-exposed and unexposed offspring.

There were 3 studies with available BMI Z score data, two of which focused on the PE group only, and one included both GH and PE. As shown in **Figure 2**, the heterogeneity of the results for PE offspring was moderate(*I* ^2^ = 65%). The mean difference in BMI Z score between exposed and control groups was insignificant (MD=-0.04, 95%CI: -0.25, 0.17). For offspring exposed to PE or GH, the heterogeneity was moderate (*I* ^2^ = 70%), and the mean difference in BMI Z score between groups was also insignificant (MD=0.00, 95%CI: -0.15, 0.15) (**Figure 3**).

**Figure 4:**
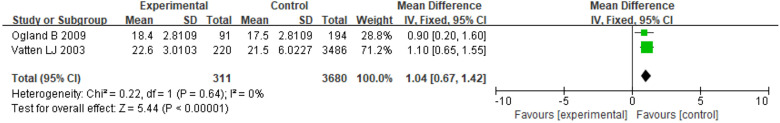
Forest plot for the mean difference in BMI between PE-exposed and unexposed female offspring.

Only 2 studies with BMI data met the criteria for meta-analysis, and the offspring of both studies were female and over 10 years old. The heterogeneity was low (*I^2^ =*0%). The results showed that the PE-exposed group had significantly higher BMI compared to the normotensive group (MD=1.04, 95% CI: 0.67~1.42, *P* < 0.05) (**Figure 4**). One study adjusted for birth weight, gestational age at birth, and age at survey attendance, and the other study adjusted for the Z score of weight for gestational age.

## Discussion

A total of 16 studies were included in our study, 15 of which were evaluated as high quality. In all offspring, GH/PE was found to be not or inversely associated with offspring adiposity outcomes in the early life (28 days-36 months) and became positively associated at larger ages (3-19 years old). In offspring with adverse birth outcomes, GH/PE-exposed group had a lower weight in the short term (28 days to 18 months), but there was a trend of rapid weight gain as they grew older, compared with the non-exposed group. Although the results of obesity prevalence were inconsistent, studies on growth trajectories also found a trend toward obesity in GH/PE offspring. Subgroup analyses indicated that the association might differ in different types of HDP or different genders. The meta-analysis supported that maternal PE exposure was associated with obesity in female offspring. There were no significant results for BMI Z score, which may be related to the large heterogeneity (*I^2 =^* 70%). And although the study of Palma Dos Reis CR et al. adjusted for confounding factors, it did not analyze important factors such as birth weight and gestational age, which may also be the reason for the biased results. Although the BMI indicator was inferior to the BMI Z score, after adjusting for covariates when analyzing BMI, we considered the conclusions to be suggestive.

Although adverse birth outcomes might partly mediate the association of GH/PE with rapid weight gain, GH/PE itself could have other mechanisms underlying the incidence of offspring adiposity. Offspring exposed to PE mothers are associated with adverse birth outcomes (e.g. SGA, IUGR) (37), and catch-up growth is often observed in these offspring (40). For example, in the study by Gow ML et al., the PE group had a higher proportion of SGA and higher weight gain than the control group. However, in the study of Washburn L et al., the association between *de novo* HDP and greater adiposity was robust after adjustment for birth weight Z score (38). HDP was related to dysregulated maternal inflammatory responses to pregnancy and could reduce the transportation of nutrients and oxygen to the fetus. According to the DOHaD, suboptimal fetal nutrition may have an epigenetic change, and the fetus would undergo adaptive physiological changes (41, 42). And by microarray analysis, PE was found to be associated with the up-regulated obese gene (43). Although in the early stage, the difference in gene expression was not significant, it would start to exert a long-term impact on adiposity outcomes.

Current evidence suggested that GH and PE might influence offspring adiposity outcomes through different mechanisms. They may have different pathophysiological effects on endothelial function, placental function, and subsequent programming of fetal growth and development (44). Different adverse birth outcomes of GH or PE also supported their different pathologic mechanisms. Researchers pointed out that PE significantly increased the risk of adverse outcomes such as placental abruption, SGA, 5 min Apgar score < 7 but GH increased the risk of preterm birth (45). In addition, a cohort study identified different serum cytokine profiles for GH and PE (46). Further research is needed to confirm the different impacts of these two diseases on offspring adiposity. For PE of varying severity, evidence showed that the inconsistent results may be related to the levels of IGF-1, which would influence prematurity and inflammation (47). In addition, early PE (before 34 + 0 weeks) and late PE (after 34 + 0 weeks) were considered distinct diseases as they were caused by different hemodynamic states (48).

In different genders, GH/PE may have different associations with offspring adiposity outcomes. Some studies have found the interaction effects of gender with GH/PE on childhood obesity. For explanation, first, different genders were considered to hold different sensitivity to tolerance to the environment and pregnancy outcomes. The male gender has been identified in many studies as an independent risk factor for adverse childbirth outcomes (49–51). Therefore, male infants exposed to GH/PE may have a more significant response. Second, altered placental function leads to related hormonal changes, including decreased estrogens, and increased progesterone and androgens (52). These sex hormones play different roles in different genders, leading to differences in growth and development. Furthermore, fetal exposure to glucocorticoids plays an important role in postpartum reproductive development (53). The adverse fetal environment, including HDP, can cause glucocorticoid disorders by regulating the expression of 11β-hydroxysteroid dehydrogenase (11β-HSD) in the placenta (54). In animal experiments, it was found that females had a greater response to glucocorticoids than males (55). In addition, leptin levels in fetuses and children also exhibit sexual dimorphism (56). The combination of gender-different susceptibility, complex hormonal changes, or other underlying factors can influence the growth trends. More research is needed to clarify the specific mechanisms and provide important implications for gender-specific interventions.

The control of confounding factors is important to the credibility of research conclusions. The studies included in our meta-analysis ensured that the results were adjusted for important factors. However, this criterion did not present in the previous systematic review (24). In the early life period, maternal factors and the state of birth can play an important role in the appearance of offspring adiposity outcomes. Maternal pre-pregnancy BMI, smoking status, parity, gestational diabetes, maternal tobacco usage during pregnancy, and maternal socioeconomic status are strong confounders of the association between maternal GH/PE and childhood obesity (30, 33). In the study by Gow ML et al., there was a lower rate for PE mothers to be breastfeeding at discharge and 6 months (27), indicating potential influence caused by breastfeeding. Especially in the poor area with lower socioeconomic strata, vulnerable infants are less likely to be breastfed than in other more developed areas (57). As children grow older, their weight can be highly influenced by lifestyle, which was also an important factor to be controlled for exploring the association between maternal GH/PE and childhood obesity (58). Differences in controlling for confounding factors among studies are also one of the reasons for inconsistent findings.

The findings of this study have important implications for the management of obesity in offspring. Maternal GH/PE-exposed offspring were observed to have more adverse birth outcomes and these offspring would present with significant catch-up growth. Although catch-up growth has short-term benefits, allowing newborns to erase the growth deficit and reduce hospital admission rates and mortality (40), it is related to long-term negative consequences such as obesity, hypertension, dyslipidemia, and insulin resistance in adulthood (59–61). Balancing the short-term benefits and adverse long-term outcomes of catch-up growth is important. Although the mechanism is unclear, prevention of GH/PE may be beneficial in alleviating childhood obesity. Thus, special attention should be paid to high-risk pregnant women, such as those with advanced age, obesity, or a history of HDP. Furthermore, for offspring exposed to GH/PE, it is important to start the management of weight growth at an early age.

Our research has three strengths. First, we used relatively strict inclusion and exclusion criteria to include high-quality studies. Studies that included women with pre-existing hypertension or those with unknown disease classification were excluded. And the type of study was strict to the cohort study, which guaranteed the quality of evidence to some extent. Second, previous systematic reviews only analyzed BMI, which could not comprehensively represent the obesity status of children (24). In this study, the outcome indicators were diverse. In addition to the main adiposity indicators (weight, BMI, etc.), we also summarized the results of other indicators (triceps skinfold thickness, waist-hip ratio, etc.). Third, we limited the observation age range. The outcome of the offspring observed in this study was 0-18 years old (only one article included a small number of 19-year-olds). This stage is the most important growth and development period in the entire life stage and is an important window for intervention.

However, this study has limitations. Few existing studies met the selection criteria, so it was difficult to have large amounts of data and perform subgroup analysis. The observation age also lacked continuity and a limited number of publications made it hard to confirm the influence caused by age heterogeneity. In addition, the control of confounding factors in different studies was inconsistent.

Future researchers could extend more in this field to provide more reliable evidence. First, the follow-up time can be extended. The study by Gow ML et al. did not observe a significant difference between the PE group and the controls, but there was a rapid growth trend, suggesting that the difference might turn out to be significant if follow-up continues (37). Second, in the future, the clinical system for pregnant women should be further improved. Many retrospective cohort studies were based on hospital case data. Although some studies had a large sample size, they lacked accurate diagnosis and treatment information. Third, observation indicators should be diversified. BMI Z score was thought to be more representative of childhood obesity than BMI. More research should be conducted using the BMI Z score for further analysis in the future. It was also recommended to supplement waist to height ratio, skinfold thickness, and other information or apply better measures, such as dual-X-ray absorptiometry in future studies to comprehensively evaluate the obesity status (29). Fourth, since different ages and races may have different results(for example, Indians tend to have lower lean body mass), studies in different populations are necessary to confirm the findings (62).

## Conclusion

The systematic review suggested that maternal exposure to *de novo* HDP was associated with obesity in offspring, extending from early childhood to adolescence. The meta-analysis showed that PE was associated with higher BMI in female offspring. More studies are needed to conduct stratified analyses by PE/GH, the severity of HDP, or gender. The future exploratory study should expand to examine the effectiveness of the prevention of *de novo* HDP in controlling the rapid growth of offspring adiposity.

## Data availability statement

The original contributions presented in the study are included in the article/**Supplementary Material**. Further inquiries can be directed to the corresponding authors.

## Author contributions

SY and JL contributed to the literature search, screening, writing, and proofreading of the manuscript. ZL helped to settle disagreements in screening the studies. SZ helped to revise and proofread the manuscript. YJ and HW designed the study, and reviewed and edited the manuscript. All authors contributed to the article and approved the submitted version.

## Funding

This study was sponsored by the Beijing Natural Science Foundation (7212144) and the National Natural Science Foundation of China (92046019). Shuang Zhou was supported by the China Scholarship Council at the Erasmus University Medical Centre, Rotterdam, The Netherlands (202106010220) as well as the Innovation Fund for Outstanding PhD Candidates of Peking University Health Science Centre.

## Conflict of interest

The authors declare that the research was conducted in the absence of any commercial or financial relationships that could be construed as a potential conflict of interest.

## Publisher’s note

All claims expressed in this article are solely those of the authors and do not necessarily represent those of their affiliated organizations, or those of the publisher, the editors and the reviewers. Any product that may be evaluated in this article, or claim that may be made by its manufacturer, is not guaranteed or endorsed by the publisher.
