# Supplementary material for: Association of gestational hypertension and preeclampsia with offspring adiposity: A systematic review and meta-analysis
Source: Front Endocrinol (Lausanne). 2022 Aug 23;13:906781. doi: 10.3389/fendo.2022.906781 (PMC9445980; doi:10.3389/fendo.2022.906781)
Supplement: Supplementary file 1 [file Table_1.docx]

**Supplementary Table 1. The detailed search strategy of the systematic review**

| Pregnancy | 1Pregnancy | [MeSH Terms] |
| --- | --- | --- |
|  | 2Pregnan* | [Title/Abstract] |
|  | 3Gestation* | [Title/Abstract] |
|  | 4Maternal | [Title/Abstract] |
|  | ① 1 OR 2 OR 3 OR 4 |  |
| Preeclampsia | 5Pre-Eclampsia | [MeSH Terms] |
|  | 6Pre Eclampsia | [Title/Abstract] |
|  | 7Preeclampsia | [Title/Abstract] |
| Gestational Hypertension | 8Hypertension, Pregnancy-Induced | [MeSH Terms] |
|  | 9hypertension during pregnancy | [Title/Abstract] |
|  | 10Hypertension, Pregnancy Induced | [Title/Abstract] |
|  | 11Pregnancy-Induced Hypertension | [Title/Abstract] |
|  | 12Pregnancy Induced Hypertension | [Title/Abstract] |
|  | 13Hypertensions, Pregnancy Induced | [Title/Abstract] |
|  | 14Induced Hypertension, Pregnancy | [Title/Abstract] |
|  | 15Induced Hypertensions, Pregnancy | [Title/Abstract] |
|  | 16Gestational Hypertension | [Title/Abstract] |
|  | 17Hypertension, Gestational | [Title/Abstract] |
|  | 18Transient Hypertension, Pregnancy | [Title/Abstract] |
|  | 19Hypertension, Pregnancy Transient | [Title/Abstract] |
|  | 20Pregnancy Transient Hypertension | [Title/Abstract] |
|  | ② 5 OR 6 OR ....OR 18 OR 19 OR 20 |  |
| offspring | 21Infant, Newborn | [MeSH Terms] |
|  | 22Adolescent | [MeSH Terms] |
|  | 23Pediatrics | [MeSH Terms] |
|  | 24infant* | [Title/Abstract] |
|  | 25newborn* | [Title/Abstract] |
|  | 26neonat* | [Title/Abstract] |
|  | 27child* | [MeSH Terms] |
|  | 28teen* | [Title/Abstract] |
|  | 29offspring* | [Title/Abstract] |
|  | 30youth* | [Title/Abstract] |
|  | ③ 21 OR 22 OR 23 OR … OR 29 OR 30 |  |
| Outcomes | 31Body Weight | [MeSH Terms] |
|  | 32adiposity | [MeSH Terms] |
|  | 33adipose | [Title/Abstract] |
|  | 34weight* | [Title/Abstract] |
|  | 35body mass index | [MeSH Terms] |
|  | 36BMI | [Title/Abstract] |
|  | 37obesity | [MeSH Terms] |
|  | 38obese | [Title/Abstract] |
|  | 39overweight | [MeSH Terms] |
|  | 40waist circumference | [Title/Abstract] |
|  | 41body composition | [MeSH Terms] |
|  | 42Waist-Hip Ratio | [MeSH Terms] |
|  | 43Body fat | [Title/Abstract] |
|  | 44Hip circumference | [Title/Abstract] |
|  | 45WFL | [Title/Abstract] |
|  | 46weight for length | [Title/Abstract] |
|  | ④ 31 OR 32 OR 33 OR …45 OR 46 |  |
| Study type | 47cohort | [MeSH Terms] |
|  | 48retrospective | [Title/Abstract] |
|  | 49prospective | [Title/Abstract] |
|  | 50longitudinal | [Title/Abstract] |
|  | 51follow up | [Title/Abstract] |
|  | ⑤ 47 OR 48 OR 49 OR 50 OR 51 |  |
| NOT | 52meta-analysis | [Title/Abstract] |
|  | 53review | [Title/Abstract] |
|  | 54editorial | [Title/Abstract] |
|  | 55case report* | [Title/Abstract] |
|  | ⑥ 52 OR 53 OR 54 OR 55 |  |
|  | 1. AND ② AND ③ AND ④ AND ⑤ NOT ⑥ | |
