# Supplementary material for: Association of gestational hypertension and preeclampsia with offspring adiposity: A systematic review and meta-analysis
Source: Front Endocrinol (Lausanne). 2022 Aug 23;13:906781. doi: 10.3389/fendo.2022.906781 (PMC9445980; doi:10.3389/fendo.2022.906781)
Supplement: Supplementary file 2 [file Table_2.docx]

**Supplementary Table 2.** **The detailed NOS scores of 16 included studies**

| study | selection | | | | comparability | outcome | | | |
| --- | --- | --- | --- | --- | --- | --- | --- | --- | --- |
|  | representativeness of the exposed cohort | selection of the non-exposed cohort | ascertainment of exposure | demonstration that outcome of interest was not present at start of study | comparability of cohorts on the basis of the design or analysis | assessment of outcome | was follow-up long enough for outcomes to occur | adequacy of follow up of cohorts | quality score |
| Baulon E et al. 2005 | ☆ | ☆ | ☆ |  | ☆ | ☆☆ | ☆ |  | 7 |
| Megan L Gow et al. 2021 | ☆ | ☆ | ☆ | ☆ | ☆ | ☆☆ | ☆ | ☆ | 9 |
| Silveira RC et al. 2007 | ☆ | ☆ | ☆ | ☆ | ☆ | ☆☆ | ☆ | ☆ | 9 |
| Jiang W et al. 2021 | ☆ | ☆ | ☆ | ☆ | ☆ | ☆☆ | ☆ | ☆ | 9 |
| Randhir K et al. 2020 | ☆ | ☆ | ☆ | ☆ | ☆ | ☆☆ | ☆ | ☆ | 9 |
| Palti H et al.  1989 | ☆ | ☆ | ☆ |  |  | ☆☆ |  | ☆ | 6 |
| Huang Y et al. 2020 | ☆ | ☆ | ☆ | ☆ | ☆ | ☆☆ | ☆ |  | 8 |
| Geelhoed JJ et al. 2017 | ☆ | ☆ | ☆ | ☆ | ☆ | ☆☆ | ☆ | ☆ | 9 |
| Palma Dos Reis CR et al.2021 | ☆ | ☆ | ☆ | ☆ | ☆ | ☆☆ | ☆ | ☆ | 9 |
| Ogland B et al. 2009 | ☆ | ☆ | ☆ | ☆ | ☆ | ☆☆ | ☆ | ☆ | 9 |
| Aris IM et al. 2018 | ☆ | ☆ | ☆ | ☆ | ☆ | ☆☆ | ☆ | ☆ | 9 |
| Byberg KK et al. 2017 | ☆ | ☆ | ☆ | ☆ | ☆ | ☆☆ | ☆ | ☆ | 9 |
| Washburn L et al. 2013 | ☆ | ☆ | ☆ | ☆ | ☆ | ☆☆ | ☆ | ☆ | 9 |
| Miettola S et al. 2013 | ☆ | ☆ | ☆ | ☆ | ☆ | ☆☆ | ☆ | ☆ | 9 |
| Davidesko S et al. 2020 | ☆ | ☆ | ☆ |  | ☆ | ☆☆ | ☆ | ☆ | 8 |
| Vatten LJ et al. 2003 | ☆ | ☆ | ☆ | ☆ | ☆ | ☆☆ | ☆ | ☆ | 9 |
